# Multi-annual performance evaluation of laboratories in post-mortem diagnosis of animal rabies: Which techniques lead to the most reliable results in practice?

**DOI:** 10.1371/journal.pntd.0009111

**Published:** 2021-02-05

**Authors:** Emmanuelle Robardet, Alexandre Servat, Jonathan Rieder, Evelyne Picard-Meyer, Florence Cliquet

**Affiliations:** ANSES, Nancy Laboratory for Rabies and Wildlife–WHO Collaborating Centre for Research and Management in Zoonoses Control; OIE Reference Laboratory for Rabies; European Union Reference Laboratory for Rabies; European Union Reference Laboratory for Rabies Serology—Bâtiment H, Technopôle Agricole et Vétérinaire, Malzéville, France; University of Calgary, CANADA

## Abstract

Rabies diagnosis proficiency tests on animal specimens using four techniques (FAT, RTCIT, conventional RT-PCR and real-time RT-PCR) were organised over 10 years (2009–2019). Seventy-three laboratories, of which 59% were from Europe, took part. As the panels were prepared with experimentally-infected samples, the error rate of laboratories on positive and negative samples was accurately estimated. Based on fitted values produced by mixed modelling including the variable “laboratory” as a random variable to take into account the longitudinal design of our dataset, the technique that provided the most concordant results was conventional RT-PCR (99.3%; 95% CI 99.0–99.6), closely followed by FAT (99.1%; 95% CI 98.7–99.4), real-time RT-PCR (98.7%; 95% CI 98.1–99.3) and then RTCIT (96.8%; 95% CI 95.8–97.7). We also found that conventional RT-PCR provided a better diagnostic sensitivity level (99.3% ±4.4%) than FAT (98.7% ±1.6%), real-time RT-PCR (97.9% ±0.8%) and RTCIT (95.3% ±5.1%). Regarding diagnostic specificity, RTCIT was the most specific technique (96.4% ±3.9%) followed closely by FAT (95.6% ±3.8%), real-time RT-PCR (95.0% ±1.8%) and conventional RT-PCR (92.9% ±0.5%). Due to multiple testing of the samples with different techniques, the overall diagnostic conclusion was also evaluated, and found to reach an inter-laboratory concordance level of 99.3%. The concordance for diagnostic sensitivity was 99.6% ±2.0% and for diagnostic specificity, 98.0% ±8.5%. Molecular biology techniques were, however, found to be less specific than expected. The potential reasons for such findings are discussed herein. The regular organisation of performance tests has contributed to an increase in the performance of participating laboratories over time, demonstrating the benefits of such testing. Maintaining a high-quality rabies diagnosis capability on a global scale is key to achieving the goal of eliminating dog-mediated human rabies deaths. The regular organisation of exercises on each continent using selected local strains to be tested according to the local epidemiological situation is one factor that could help increase reliable diagnosis worldwide. Rabies diagnosis capabilities could indeed be enhanced by providing adequate and sustainable proficiency testing on a large scale and in the long term

## Author summary

This study shares the rabies diagnosis proficiency test results of 73 laboratories on animal specimens using four techniques (FAT, RTCIT, conventional RT-PCR and real-time RT-PCR) organised over a 10-year period. This long-term exercise allowed us to compute accurate sensitivity and specificity values for the rabies diagnosis test for a large panel of laboratories. Conventional RT-PCR provided a better diagnostic sensitivity level than FAT, real-time RT-PCR and RTCIT. Regarding diagnostic specificity, RTCIT was the most specific technique followed closely by FAT, real-time RT-PCR and conventional RT-PCR. The specificity of molecular biology techniques was found to be lower than expected. The potential reasons for such findings are discussed herein. The regular organisation of performance tests has contributed to an increase in the performance of participating laboratories over time, demonstrating the likely benefits of such testing.

## Introduction

Effective disease surveillance is vital in order to obtain an accurate estimation of an epidemiological situation and consequently implement appropriate control measures. Surveillance systems are mainly based on tests on an individual scale. To quickly and effectively detect disease outbreaks, sensitive and specific tests are necessary [1]. In terms of precision of measurements, the good repeatability (within-day variability) and intermediate precision (day-to-day variability) of results are also important to allow comparable estimation over time [2]. A distinction must be made between analytical and diagnostic characteristics. Analytical sensitivity estimates the test’s limit of detection, and analytical specificity assesses the test’s ability to distinguish the target analyte from non-target analytes [3]. On the other hand, diagnostic sensitivity estimates the probability that a truly infected individual will be classified as infected using the test, and diagnostic specificity is the probability that a healthy animal will be classified as healthy using the test [3]. Such performance characteristics are often estimated during the technique’s validation process and its implementation in a laboratory [4]. In a situation where diseases occur over large areas, several laboratories may be included in the diagnosis network. It is of paramount importance to be able to compare results in space (within different states, regions or districts, etc.) and in time (at different periods of the year or in different years). In such circumstances, inter-laboratory assessments can be used to ensure the appropriate reproducibility of results. Once such measures are in place, inter-laboratory tests can be regularly organised on a long-term basis to ensure that the laboratories involved in testing produce valid and comparable results over time. This is the objective of proficiency tests [5], which therefore assess the performance of a laboratory and not of the technique itself.

Rabies is a life-threatening disease infecting some 59,000 people every year worldwide [6]. All mammals are susceptible to rabies, but dogs are the most common animal involved, with more than 99% of human rabies cases being the direct result of dog bites [7]. It is also a longstanding disease, as the first reports appear to date back to antiquity [8]. This zoonosis has been preventable since 1885, when Louis Pasteur first inoculated a 9-year-old dog-bite victim with the dried brains of a rabid rabbit [9]. Effective vaccines for both humans and dogs are currently available on the market [7]. By the 1980s, the disease had essentially been eliminated from dogs and people in many developed countries, but half of the world’s population still lives in countries where rabid dogs threaten their lives due to poor economic conditions [10]. Moreover, wildlife constitutes a rabies reservoir in some parts of the world (e.g. North America, Eastern Europe) and potential spill-over remains a real health risk. Rabies is one of the major neglected diseases. Multiple reasons could explain this tragic situation [11,12]: the comparatively low economic impact due to the dog being the most affected species, lack of awareness among policy-makers of the rabies situation and its impacts, lack of awareness among the population, and the limited interest shown by health authorities.

As the disease has long been present on all continents except Antarctica, rabies diagnostic networks are extended. Among others, there are 13 WHO (World Health Organization) Collaborating Centres https://apps.who.int/whocc/List.aspx?tor=rabies& and 11 OIE (World Organisation for Animal Health) reference laboratories https://www.oie.int/en/scientific-expertise/reference-laboratories/list-of-laboratories/. For the European Union, just as an example, there are 25 National Reference Laboratories (NRLs) https://eurl-rabies.anses.fr/en/minisite/rabies/eu-national-reference-laboratories-rabies supported by numerous regional laboratories. Proficiency testing helps to ensure the comparability of results within such extended networks. As the OIE and European Union Reference Laboratory (EURL) (Commission regulations No. 737/2008 and No. 415/2013 [13]), the ANSES Nancy Laboratory for Rabies and Wildlife has organised annual inter-laboratory tests for animal rabies post mortem diagnosis since 2009 for the benefit of European National Reference Laboratories (NRLs), OIE and WHO laboratories, and some voluntary laboratories from third countries.

ISO/IEC 17025 is the International Organization for Standardization (ISO) standard used in laboratories to provide a basis for accreditation of laboratory quality systems [14]. Since its first release in 1978 and subsequent editions in 1982 and 1990, the standard of quality assurance system management has been implemented in many laboratories worldwide. ISO 17025 defines proficiency testing as the “evaluation of participant performance against pre-established criteria by means of inter-laboratory comparisons” and is indeed a good means for laboratories to demonstrate their ability to meet the desired level of quality. According to ISO 17025, accredited testing and calibration laboratories are required to participate in proficiency testing programmes. Laboratories that participate in such tests can therefore be accredited for certain techniques but their participation also helps maintain their accreditation status, motivating them to pursue participation. In certain areas of laboratory expertise, like serological testing of pets in the framework of travel schemes to certify proper rabies vaccination, for example, proficiency tests can even lead to institutional designation of officially-approved laboratories for testing [15].

This paper describes 10 years’ participation by 73 worldwide national laboratories in animal rabies post-mortem diagnosis proficiency trials assessing the fluorescent antibody test (FAT), the rabies tissue culture infection test (RTCIT), conventional reverse transcription polymerase chain reaction (RT-PCR) and real-time RT-PCR laboratory competencies. Scientific literature classically reports the sensitivity and specificity of the techniques assessed and published through a validation process performed by a single laboratory, but such an assessment does not provide information on the accuracy of the rabies diagnostic network in itself. This study presents a large scale and regular assessment of laboratory performance over time, thus providing a comprehensive and robust overview of the diagnostic quality results of laboratories involved in rabies surveillance on various continents.

## Materials and methods

### Ethical statement

Animal experiments to produce rabies-positive brain tissues complied with regulation 2010/63/EC of the European Parliament and the council of 22 September 2010 on the protection of animals used for scientific purposes [16] and as transposed into French law [17]. These experiments were approved by the ANSES/ENVA/UPEC ethics committee and the French Ministry of Research (Apafis n°11772–2017101311312783). Institutional and national guidelines for the care and use of laboratory animals were strictly followed to ensure appropriate protocols and welfare conditions.

### Characteristics of participating laboratories over time

From 16 (in the RTCIT trial of 2009) to 50 national laboratories (in the FAT trial of 2016) participated annually in the proficiency test. Globally, over the 10 years of the study period, 73 different laboratories participated, of which 59% were from Europe (including 38% from the European Union (EU)), 17% from Africa, 12% from Asia, 11% from the Americas and 1% from Oceania. Considering the regular and repeated participation from one year to another, the representativity of participants over the five continents was 55% from the EU, 18% from the non-EU part of Europe, 13% from Africa, 7% from the Americas, 5% from Asia and 2% from Oceania. The evolution in participants per continent over the years is shown in Table 1. More than 70% of the total number of laboratories participating yearly in the proficiency testing (PT) were from Europe. Because participation in diagnosis proficiency tests organised by the EURL is mandatory, regulatory and free of charge for NRLs within the EU, many study participants are from the EU (Commission regulations No. 737/2008 and No. 415/2013 [13]).

**Table 1:** Evolution of the number of laboratories participating in proficiency testing for rabies diagnosis per continent.

Continent	2009	2010	2011	2012	2013	2014	2015	2016	2017	2019
EU	22 (69%)	25 (60%)	24 (57%)	25 (54%)	25 (58%)	25 (50%)	25 (53%)	26 (49%)	25 (53%)	26 (55%)
Europe (non-EU part)	4	6	7	6	10	10	8	12	8	11
Total Europe (% of the total)	26 (81%)	31 (74%)	31 (74%)	31 (67%)	35 (81%)	35 (70%)	33 (70%)	38 (72%)	33 (70%)	37 (79%)
Africa	3	5	6	7	3	8	6	8	6	4
Americas	1	2	2	6	4	4	5	1	4	3
Asia	1	3	2	1	0	2	2	5	3	2
Oceania	1	1	1	1	1	1	1	1	1	1
Total worldwide	32	42	42	46	43	50	47	53	47	47

### Panel composition and preparation

Test panels were composed of 8–10 coded samples. A single panel was sent each year to test the different techniques routinely used in the participating laboratory as part of its animal rabies post-mortem diagnostic process. All the participants received the same panel but with samples coded differently. Samples contained 1 mL of lyophilised brain homogenate of mouse, dog, pig or raccoon dog origin experimentally infected by various fixed (CVS-27) or field strains of lyssavirus such as RABV (*Rabies lyssavirus*), EBLV-1 (*European bat lyssavirus 1*), EBLV-2 (*European bat lyssavirus 2*), DUVV (*Duvenhage lyssavirus*), BBLV (*Bokeloh bat lyssavirus*) or ABLV (*Australian bat lyssavirus*). At least one RABV and one EBLV-1 sample were included each year in the test (Table 2). Negative samples (NEG) originated from confirmed negative red fox or pig brain samples from France (a country free from infection with RABV). Some batches named “RABV dil” were RABV samples (from a French RABV GS7 strain isolated in 1990) diluted with a rabies-negative brain sample. All the ABLV, BBLV and DUVV batches used during the study were produced from a single unique virus strain (strain 127900 for BBLV, strain 96132 for DUVV and strain 96/0648 for ABLV). EBLV-2 was from strain RV1332 from 2009 to 2012, and then from RV1787 from 2013 to 2019. EBLV-1 was from EBLV-1a (strain 122936) or EBLV-1b (strain 121411 from 2009 to 2012 and strain 123008 from 2013 to 2019) (see S1 Table). RABV batches were from different rabies viruses isolated in various parts of the Maghreb or Europe (See S2 Table). Various additional samples from previous batches were also included in panel tests (from 2016 to 2019) in order to vary the composition of panels from one participant to another and thus avoid collusion between laboratories. These samples were not considered for the evaluation.

Virus batches were produced *in vivo* according to Robardet et al. (2011) [18]. Briefly, mice, foxes, dogs or raccoon dogs were inoculated intracerebrally. Animals having shown signs suggestive of rabies (experimentation continued up to stage 4/5 of the disease [19]) validated *a posteriori* by FAT diagnosis [20,21] were humanely euthanised. The first animal of a batch production with suggestive signs was diagnosed by the FAT to confirm rabies infection. For each batch of virus, the brain samples from euthanised animals were excised then mixed, homogenised and aliquoted into 1 mL tubes before being freeze-dried. Whenever possible, brain samples of foxes, dogs and raccoon dogs were collected from other scientific research being carried out in the laboratory as long as the virus inoculation protocol was the same as that used for virus production dedicated to rabies diagnosis proficiency test studies. Panel samples were the lyophilised homogenates of fresh infected brains, each sample representing different viral strains and all blindly coded.

**Table 2:** Lyssavirus species and negatives used in panels over the years.

Virus Species	2009	2010	2011	2012	2013	2014	2015	2016	2017	2019
ABLV		X	X		X				X	X
BBLV							X	X		
DUVV				X			X			X
EBLV-1	X	X	X	X	X	X	X	X	X	X
EBLV-2	X	X	X	X	X	X	X	X	X	
NEG	X	X	X	X	X	X	X	X		X
RABV	X	X	X	X	X	X	X	X	X	X
RABV dil	X		X	X		X		X		

### Panel stability

A panel is considered stable when, under different conditions of temperature and storage duration (conditions that can be induced during the study), the final result is the same as the initial result. After production, lyophilised samples were stored at 5±3°C. From the authors’ own experience, lyophilised panels produced in the laboratory and stored at 5±3°C remain stable for at least 3 years (personal communication). The panels of samples were therefore shipped to participating laboratories at 5±3°C, in accordance with the international regulation on diagnosis samples and using a specialist carrier for diagnostic samples (UN3373 conditions) [22]. In agreement with the specifications of the ISO 13528 International Standard [23], sample stability was evaluated blindly on coded samples just before each dispatch of the samples selected for the annual proficiency test. At least two main conditions were assessed annually: samples kept for 7 and 14 days at room temperature to mimic long shipping periods and improper temperature conditions very different from the 5±3°C requested for the shipment. The objective of this stability study was to estimate the time during which positive samples remained positive and negative samples remained negative and without artefacts caused by any bacteria that could have grown under such improper temperature conditions during storage [24]. When the shipping time exceeded the sample validity time, the laboratory results of the considered samples were excluded from the evaluation.

### Panel homogeneity

The homogeneity of each batch produced was tested after the lyophilisation step. Each technique proposed in the trial was assessed to analyse several samples chosen at random from the batch production according to ISO 13528: three samples from 2009 to 2013, and then ten samples (since 2012 for negative batches and since 2014 for positive batches) were analysed using all the techniques included in the proficiency test to ensure homogeneity, meaning that positive samples all tested positive and negative samples all tested negative. All the batches used in this study were validated accordingly. One negative batch was excluded after the proficiency test had been carried out due to later detection of the presence of RNA contamination. This batch was used in 2017 and excluded from the evaluation and not therefore included in this study.

### Testing of samples by participants

Proficiency tests were organised on an annual basis from 2009 to 2017. No proficiency test was organised in 2018 as it was decided to hold the trial every 2 years from 2017. Upon registration, each participating laboratory had to choose the tests it wanted to apply out of FAT, RTCIT [2,21,25,26], real-time RT-PCR [2,21,27–29] or conventional RT-PCR [2,21,30] and the mice inoculation test (MIT) [2,21,31]. MIT was assessed in 2009 only and real-time RT-PCR has been assessed since 2011 with the exception of 2017. The participating laboratories had to evaluate the tests they were using routinely according to their routine protocols, so no protocols were consequently sent with the samples. As multiple diagnostic tests were assessed on the same samples (FAT and RTCIT, or FAT, RTCIT and conventional RT-PCR, or FAT and real-time RT-PCR, etc.) a global diagnostic conclusion was also requested per sample from 2017 onwards to mimic as closely as possible the usual conditions under which rabies is diagnosed, i.e. for each sample, all the laboratories were invited to conclude on the animal’s infection status (infected if positive/not infected if negative) on the basis of their results.

The laboratories were advised to store lyophilised samples at 5±3°C upon their reception, and to perform the tests as soon as possible thereafter. Each laboratory had to check the samples’ condition on arrival and to fill out an acknowledgement of receipt. If panels arrived damaged or in a suspect condition, they could be replaced upon the participating laboratory’s request. Because freeze-dried samples were sent, they had to be reconstituted by adding 1 mL of sterile distilled water under a biosafety cabinet. After adding water, a 30-minute waiting period was necessary to rehydrate the brain homogenates. Each participant had to state on the result form the results for each sample of each technique evaluated (positive or negative, detected or not detected). Furthermore, in order to allow a fair evaluation of participants, each laboratory had to state which strains the laboratory technique was able to detect. In this way, any wrong results for strains that the laboratory’s techniques were not supposed to amplify were not taken into consideration.

### Evaluation criteria

Laboratory performances were assessed in agreement with the ISO 13528 standard, by considering the number of concordant, i.e. correct, results from qualitative testing. The result of a participating laboratory for a considered test (FAT, RTCIT, real-time RT-PCR, conventional RT-PCR) was considered “correct” when the laboratory detected the presence or absence of the lyssavirus in the positive and negative samples respectively (lyssavirus antigen for FAT, infectious live lyssavirus for RTCIT, lyssavirus RNA for conventional RT-PCR and real-time RT-PCR). A result was defined as “incorrect” when a discordant result occurred for a considered test (positive sample not found positive or negative sample not found negative). The evaluation of the diagnostic conclusion was considered “correct” if the diagnostic conclusion for the sample was concordant with the sample’s disease status. If an incorrect result occurred, the diagnostic conclusion was considered “incorrect”.

### Statistical analysis

Statistical analyses were carried out using R software version 3.5.2 [32]. The level of performance (proportion of concordant results irrespective of the sample’s negative or positive status) was evaluated for each laboratory, for each test, for each year and also by lyssavirus species to provide a global view of the results. Diagnostic sensitivity (the proportion of true positive samples out of the total number of experimentally infected samples i.e. the proportion of positive samples identified correctly) and diagnostic specificity (the proportion of true negative samples out of the total number of negative samples i.e. the proportion of negative samples identified correctly) were calculated for each laboratory. A mean diagnostic sensitivity for laboratories was assessed per technique (FAT, RTCIT, MIT, conventional RT-PCR, real-time RT-PCR) and per viral species (ABLV, BBLV, DUVV, EBLV-1, EBLV-2, RABV, RABV dil) while a mean diagnostic specificity was assessed per technique for each laboratory.

In order to estimate an accurate diagnostic sensitivity and specificity for rabies diagnostic tests, the proficiency test results were analysed using a binomial distribution per test (FAT, RTCIT, conventional RT-PCR, real-time RT-PCR and overall diagnostic conclusion). The response variable considered was the “correct” or “incorrect” outcome of a test. As many of the same laboratories participated annually in the proficiency test over the period, the 10-year data set included non-independent observational data due to repeated participation of some laboratories. A generalised linear mixed effects model (GLMM) was thus used with the lme4 package [33,34] to combine both the theory of generalised linear models (with the binomial distributed response variable of our study) and the linear mixed effects models for repeated (longitudinal) measurements in data analysis [35]. The “laboratory” variable was consequently set as a random effect of the model. The two dependent variables considered were “Year”, including the period 2009–2019 and “Species”, including ABLV, BBLV, DUVV, EBLV-1, EBLV-2, RABV, RABV dil and negative samples. To select the most efficient model, we used the Akaike information criterion corrected for a small sample size (AICc) [36] using the MuMin package [37] to compare the models with all combinations of fixed effects keeping the same “laboratory” random effect. Wald tests were used to examine and indicate the significance (p value < 0.05) of the variables retained in the final model [38]. Additional analyses were carried out in order to estimate the performance of each laboratory on the molecular biology techniques used most frequently by the panel of participants. This evaluation could only be carried out between 2013 and 2017, a period when information on the type of PCR technique used for the conventional and real-time RT-PCR tests were available. Thus, using the same type of model, the estimation of the sensitivity and specificity was carried out for the conventional RT-PCR using the primers of Heaton et al. [30], more specifically for the one-step conventional RT-PCR and for the two-step conventional RT-PCR. For the real-time RT-PCR, the estimation was carried out for the SYBR Green real-time RT-PCR technique and for the TaqMan real-time RT-PCR technique, all using the primers published by Wakeley et al. [27].

Receiver operating characteristic (ROC) curves can also be used to compare the diagnostic performance of two or more laboratories or diagnostic tests [39]. ROC curves were thus generated for each technique by plotting the predicted sensitivity against predicted specificity at various threshold settings based on GLMM estimators explaining the response variable as “positive” or “negative” results in tests. The same fixed effect dependent variables (“Year” and “Species”), the random effect “laboratory” and the model selection process were implemented as previously. Predicted data based on these models were then used to plot adjusted ROC curves. ROC curves evaluate performance for classification problems at various threshold settings by showing the trade-off between sensitivity (“true positive rate”; 1 - “false negative rate”) and specificity (“true negative rate”; 1 –“false positive rate”). The diagnostic technique that gives curves closer to the top left corner indicates a better performance. As a baseline, a random diagnostic technique is expected to give points lying along the diagonal of the graph. The closer the curve comes to the 45-degree diagonal of the ROC space, the less accurate the test is. The area under the curve (AUC) being a useful tool for assessing the performance of a diagnostic test over the range of possible values of a predictor variable [40], the AUC was thus calculated to compare the accuracy of the different tests, possible AUC values ranging from 0.5 (estimated as no diagnostic ability) to 1.0 (estimated as perfect diagnostic ability).

## Results

### Global frequency of result concordances

**Fig 1:**
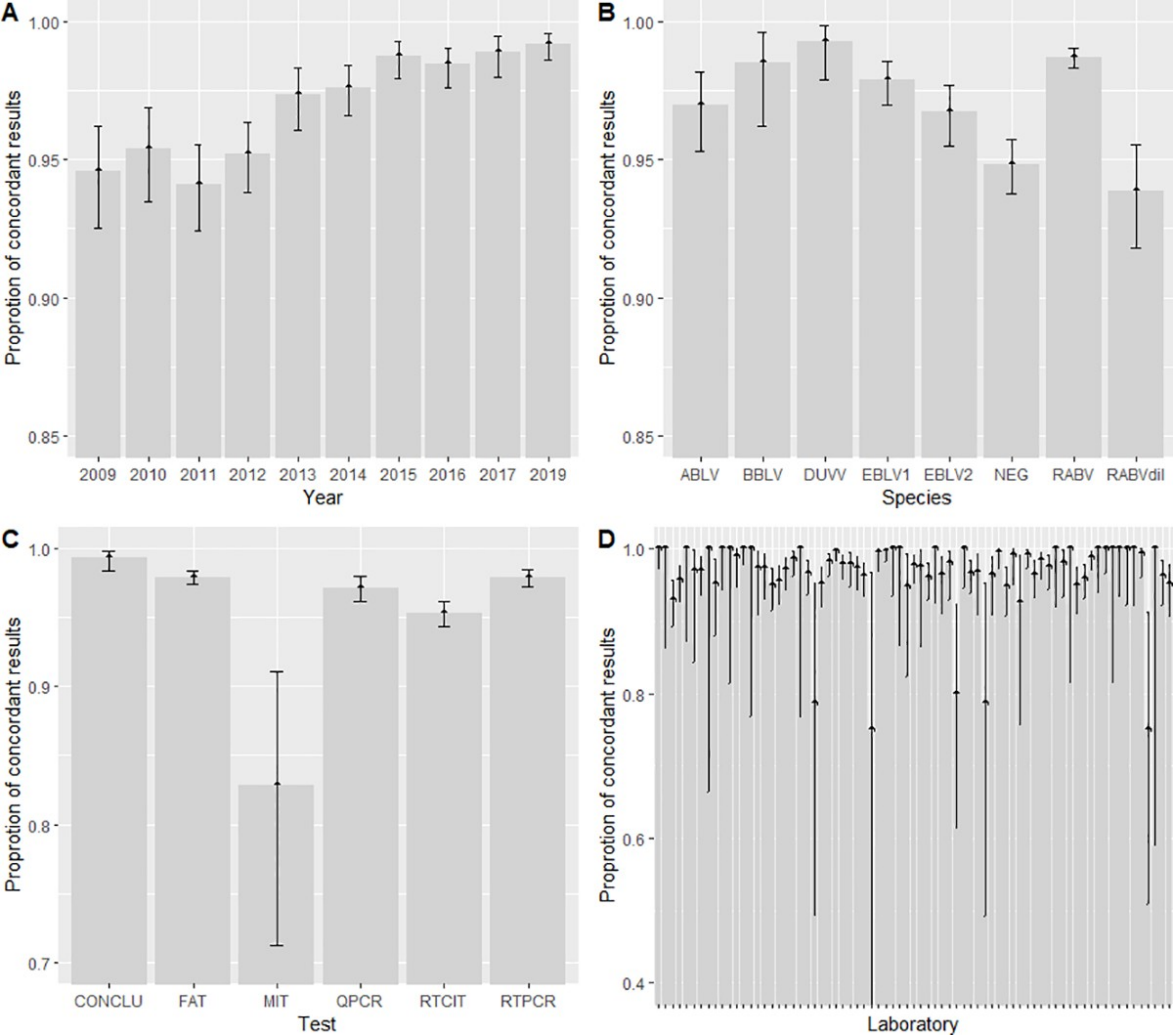
Proportion of concordant results according to year (A), test (B), species to be detected (C) and participating laboratory (n = 73). One bar represents one laboratory (D) in the 10-year dataset. Bars indicate 95-percent interval confidence of proportion. In figure B, the “QPCR” label represents real-time RT-PCR results and the “RTPCR” label represents conventional RT-PCR results.

Fig 1 presents the evolution in performance level of participating laboratories over the years. Laboratory performances (Fig 1A) rose from 94% in 2011 to 99% in 2019. This favourable evolution reveals the higher performance of participants over the years (pχ^2^ = 2.94E-19). All the techniques except MIT (83% of concordant results) and all undiluted virus samples (ABLV, BBLV, DUVV, EBLV-1, EBLV-2, RABV) had a mean proportion of concordant results over 95% (Fig 1B and 1C). As MIT was assessed in 2009 only (n = 8 participating laboratories) and because those results have already been published [18], it will not be considered in the subsequent stages of the study. When considering the viral strain species, the proportion of concordant results varied from 94% with RABV dil to 99% with DUVV. The proportion of concordant results for most of the participating laboratories (81%) was over 95%. The proportion of concordant results per laboratory varied from 75% to 100%.

### Mean diagnostic sensitivity and specificity among participating laboratories

**Fig 2:**
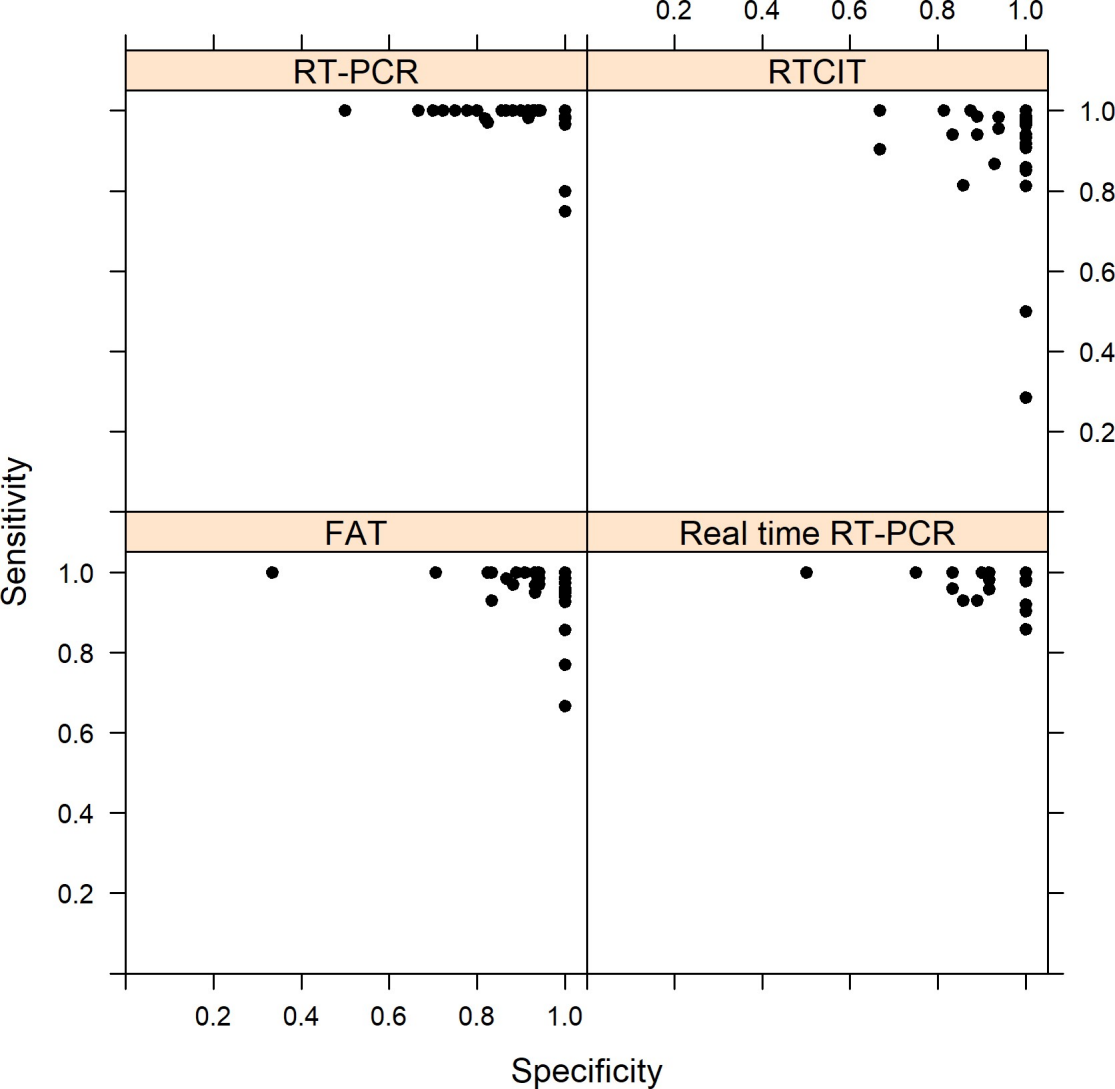
Scatterplot of sensitivity and specificity for participating laboratories (one dot: proportion for one laboratory over the 10-year study period) for each technique (FAT: 70 laboratories; RTCIT: 44 laboratories; conventional RT-PCR: 57 laboratories; real-time RT-PCR: 41 laboratories).

Sensitivity and specificity were calculated for each laboratory per test and per strain irrespective of the year (Fig 2). For FAT (n = 70 laboratories), the mean sensitivity of participating laboratories was 97.9%, ranging from 66.7% to 100% while the mean specificity was 96.1%, ranging from 33.3% to 100%. For RTCIT (n = 44 laboratories), the mean sensitivity of participating laboratories was 93.5%, ranging from 28.6% to 100% while the mean specificity was 96.0%, ranging from 66.7% to 100%. For conventional RT-PCR (n = 57 laboratories), the mean sensitivity of participating laboratories was 98.5%, ranging from 75.0% to 100% while the mean specificity was 93.6%, ranging from 50% to 100%. Finally, the mean sensitivity of participating laboratories was 98.1%, ranging from 85.7% to 100% while specificity was 94.3% ranging from 50.0% to 100% for real-time RT-PCR (n = 41 laboratories).

### Estimated diagnostic sensitivity and specificity for FAT, RTCIT, conventional RT-PCR, real-time RT-PCR and overall diagnostic conclusion

To consider non-independence of data due to the repeated participation of some laboratories over time (23/73 laboratories participated in each session, 11/73 participated only once), diagnostic sensitivity and specificity for the tests were estimated based on the mean and standard deviation of fitted data based on a logistic mixed effects model adjustment considering the variable ‘laboratories’ as a random variable (Table 3). The mean FAT sensitivity was 98.7% (SD 1.6) while specificity was 95.6% (SD 3.8) and the mean RTCIT sensitivity was 95.3% (SD 5.1) while specificity was 96.4% (SD 3.9). For conventional RT-PCR, the mean sensitivity was 99.3% (SD 4.4) while specificity was 92.9% (SD 0.5). Finally, for real-time RT-PCR, the mean sensitivity was 97.9% (SD 0.8) while specificity was 95.0% (SD 1.8). Globally, the overall diagnostic conclusion was 99.6% (SD 2.0) sensitive and 98.0% (SD 8.6) specific. The same estimations were carried out on the European participant panel only and no significant difference was observed compared to the results from the general dataset. The results of the evaluation of performance for each molecular biology technique (those most commonly used by participants) are indicated in Table 4. For the conventional RT-PCR (primer published by Heaton et al.), the estimation was carried out for one-step and two-step techniques separately. For the real-time RT-PCR techniques, the estimation was carried out for Taqman and SYBR Green techniques separately.

**Table 3:** Diagnostic sensitivity and diagnostic specificity (mean and standard deviation) estimations based on fitted data from mixed model regression (2009–2019 period).

Mean (SD)	FAT	RTCIT	conventional RT-PCR	real-time RT-PCR	Overall conclusion
Diagnosis Sensitivity	98.7 (1.6)	95.3 (5.1)	99.3 (4.4)	97.9 (0.8)	99.6 (2.0)
Diagnosis Specificity	95.6 (3.8)	96.4 (3.9)	92.9 (0.5)	95.0 (1.8)	98.0 (8.5)
Sensitivity for ABLV	98.7 (1.3)	97.0 (3.3)	99.4 (0.5)	82.2 (6.2)	99.0 (4.5)
Sensitivity for BBLV	99.1 (0.8)	98.4 (1.3)	98.7 (0.8)	98.1 (0.9)	/
Sensitivity for DUVV	100 (0)	97.5 (3.3)	99.0 (0.7)	100 (0)	100 (0)
Sensitivity for EBLV-1	98.5 (1.4)	96.7 (3.6)	99.2 (0.6)	96.3 (1.5)	100 (0)
Sensitivity for EBLV-2	98.6 (1.3)	93.4 (6.4)	97.9 (1.4)	99.3 (0.3)	100 (0)
Sensitivity for RABV	99.6 (0.4)	95.9 (4.6)	99.8 (0.1)	99.2 (0.3)	99.5 (2.5)
Sensitivity for RABV dil	93.1 (5.7)	88.9 (9.3)	99.4 (0.4)	98.4 (0.7)	/

Real-time RT-PCR since 2011 only; overall conclusion evaluated in 2017 and 2019 only; no BBLV or RABV dil included in the panel when the overall conclusion was assessed.

**Table 4:** Diagnostic sensitivity and diagnostic specificity (mean and standard deviation) estimations based on fitted data from mixed model regression for the molecular techniques most commonly used by the panel of participants (2013–2017 period only).

	conventional RT-PCR		real-time RT-PCR	
Mean (SD)	One-step	Two-step	TaqMan	SYBR Green
Diagnosis Sensitivity	99.7 (0.3)	99.2 (0.6)	97.4 (3.5)	100 (0)
Diagnosis Specificity	95.6 (4.7)	93.8 (4.0)	96.7 (4.4)	90.8 (24.1)

### Accuracy of tests and ROC curves

**Fig 3:**
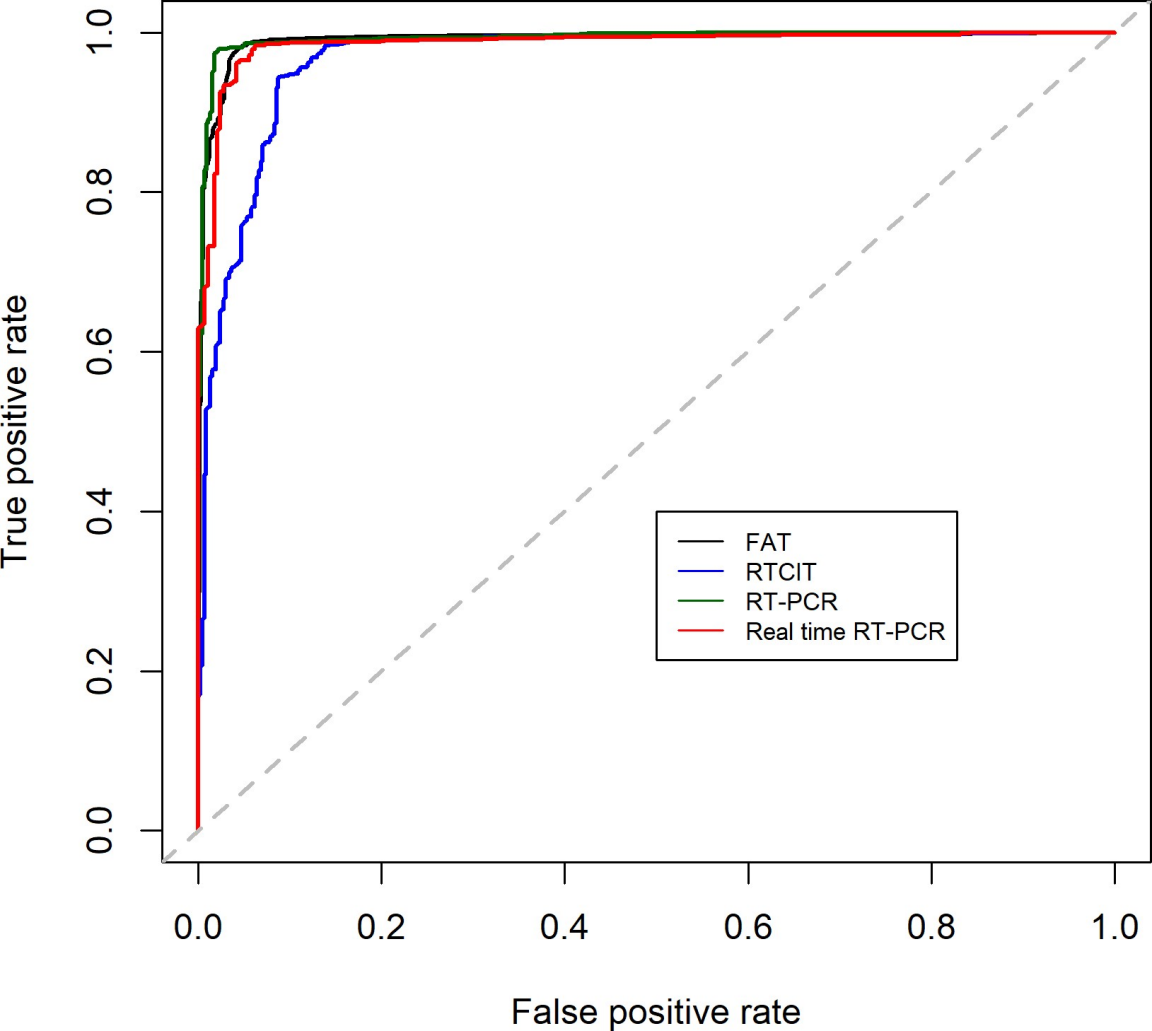
Estimated ROC plots of sensitivity against 1- specificity per rabies diagnostic technique based on mixed effects logistic regression estimators.

As indicated by the ROC curve (Fig 3), conventional RT-PCR was estimated to be the best technique for discriminating positive (infected animal) from negative (uninfected animal) samples. This is supported by AUC values of 0.991 for FAT, 0.968 for RTCIT, 0.993 for conventional RT-PCR and 0.987 for real-time RT-PCR. However, all are considered excellent predictors as the AUC reaches 0.900.

## Discussion

Diagnostic tests are imperfect, thus leading to a potential individual misclassification into healthy or diseased categories. Evaluating the degree of misclassification is of importance as it helps to assess the accuracy of epidemiological data. In the present study, we evaluated 10 years of proficiency test results involving four post-mortem animal rabies diagnostic techniques (FAT, RTCIT, conventional RT-PCR and real-time RT-PCR). Our study presents a multi-annual exercise involving a wide range of 73 international reference laboratories of which 59% were from Europe. The large number of participating laboratories from Europe reflects more a European than a worldwide view of the estimated accuracy of animal rabies diagnosis, but has the advantage of providing an estimation for a large panel and over a long period. The evaluated tests were all organised annually (since 2009 for FAT, RTCIT and conventional RT-PCR) except real-time RT-PCR, which has been organised since 2011 only. Some laboratories participated annually, while others took part less regularly. To consider the repeated participation of some laboratories we used mixed logistic models to estimate the global accuracy, sensitivity and specificity. Because these trials were based on the assessment of laboratory diagnosis of samples with a known disease status due to experimental infection, we were able to accurately estimate the diagnosis error rate for the positive and negative samples. Based on fitted values produced by mixed modelling including the variable ‘laboratories’ as a random variable to consider the longitudinal design of our dataset, the technique which provided the most concordant results was conventional RT-PCR (99.3%; 95% CI 99.0–99.6) closely followed by FAT (99.1%; 95% CI 98.7–99.4), real-time RT-PCR (98.7%; 95% CI 98.1–99.3) and then RTCIT (96.8%; 95% CI 95.8–97.7). We also found that conventional RT-PCR provided better sensitivity (99.3%) than FAT (98.7%), real-time RT-PCR (97.9%) or RTCIT (95.3%). Regarding specificity however, RTCIT was the most specific technique (96.4%) followed by FAT (95.6%), real-time RT-PCR (95.0%) and conventional RT-PCR (92.9%). The results of participating laboratories evolved favourably over time, indicating improved performance. Performance nonetheless differed according to the strain evaluated; diluted RABV samples were the positive samples leading to the highest frequency of discordant results. This is easily explained by the increased difficulty of this type of sample, as highly diluted RABV samples (weak positives) may be found to be positive or negative depending on the analytical sensitivity of the tests practised by the laboratories.

Very few proficiency test results for rabies diagnosis have been published to date. The first one presented the laboratory performance results from a FAT trial performed in 2001 in 16 European laboratories [41]. Thereafter, two trials were organised in 2009 and 2010 on a larger panel with respectively 32 and 42 participants. There was not a single discordant result for respectively 87% and 85% of the participating laboratories for FAT, 70% and 77% for RTCIT, 91 and 81% for conventional RT-PCR and 35% of laboratories for the MIT organised in 2009 only [18]. Another paper has described the results of a Latin America and Caribbean network of 23 laboratories from a FAT proficiency exercise highlighting the need to harmonise this technique with a mean concordance among participants of 81% [42]. Recently, a study presented the results of 13 sub-Saharan African laboratories taking part in FAT and conventional RT-PCR proficiency tests with respective mean laboratory concordances of 88% and 98% [43].

The rabies diagnosis proficiency testing exercises presented in the scientific literature have shown that in practice, the accuracy of diagnostic results could be lower than presented in the literature on reference standards [41–43]. The OIE manual indicates that both FAT and PCR techniques provide a reliable rabies diagnosis in 98% to 100% of cases when appropriate conjugate or primers are respectively used, and that FAT is recognised as a highly sensitive and specific technique (between 96% and 99%)[44]. Using RTCIT, Rudd et al. [45] indicated a sensitivity of 95% while Bourhy et al. [46] indicated RTCIT sensitivity and specificity (related to FAT) of 94% and 100% respectively. In our study, probably due to the long period covered, both FAT and RTCIT performance data were comparable to the ranges indicated in reference standards, even with a large panel of laboratories.

The analytical characteristics of conventional RT-PCR methods have been previously evaluated as close to 95% and 100% specific though this data vary according to the target used [30,47–50]. Real-time RT-PCR has been shown to be between ten and 1,000 times more sensitive than conventional nested RT-PCR [27,51–56]. In our study, both conventional RT-PCR and real-time RT-PCR had lower specificity than in intra-laboratory evaluations. The same observation was made for proficiency testing focusing on sub-Saharan African countries, where 89% sensitivity and 86% specificity were estimated when using conventional RT-PCR [43]. Several possibilities may explain variations of specificity in RT-PCR techniques like the primer/probe model [57], the RT-PCR system (one-step vs. two-step) [58] as well as the enzyme mix or even the thermocycler [58,59]. In our study, laboratories used mainly pan-lyssavirus primers described by Heaton et al. [30] for conventional RT-PCR and the pan lyssavirus primers described by Wakeley et al. [27] and primer/probe models described by Hoffman et al. [28] for real-time RT-PCR. Two-step hemi-nested PCR, which involves more frequent opening and handling of tubes than a one-step technique, could actually increase the non-specific amplification, resulting in false positive results. The real-time RT-PCR results of our study suggest that the SYBR Green method had a lower specificity than the TaqMan method, but greater sensitivity. The SYBR-based method being more generic, non-specific binding of the SYBR Green dye to dsDNA is indeed more likely using the SYBR Green method than using the TaqMan method, which is known to be unable to detect some species of lyssaviruses and rabies virus variants based on *in silico* analysis [27]. Moreover, the SYBR Green method requires the analysis of dissociation curves to confirm the specificity of the test result. This dissociation curve analysis can sometimes be challenging, especially when samples are autolysed or when there are PCR interferences such as inhibitors. Late C_T_ values may also generate false positive results. Finally, false positive outcomes could also have occurred in the event of cross-contamination between samples or from positive controls (two-thirds of laboratories failing in conventional RT-PCR failed one time on a single sample and never failed again, and half of laboratories failing in real-time RT-PCR also failed one time on a single sample and never failed again), which can be prevented by applying strict quality control procedures [28,60]. Such findings highlight the potential difficulty in using such highly sensitive techniques, principally on brain samples from experimentally-infected animals as used in our ring trial. The OIE recommends that pan-lyssavirus RT-PCR methods should be used as a primary rabies diagnostic test. It has been recently shown that the pan-lyssavirus real-time RT-PCR technique has much improved sensitivity and specificity in a multi-laboratory validation [61].

An evaluation of the concordance of results provides an estimation irrespective of the false positive or false negative status of the samples tested. However, in the context of rabies diagnosis, false negative results are far more harmful than false positive results, as misdiagnosis could lead to an exposed person not being treated. For this reason, both the OIE and WHO recommend that laboratories carry out a confirmation test on any sample submitted in the event of human exposure, thus limiting the occurrence of diagnostic errors [44]. In our study, FAT and conventional RT-PCR were shown to be the most accurate out of all the techniques used in this study, but by assessing the overall diagnostic conclusion, we showed that when several tests were performed, this conclusion provided by far the best accuracy. The advantage of multiple testing has already been pointed out [62]. In a multiple testing context, the diagnostic conclusion will depend on the laboratory’s decision tree, which is not currently harmonised across Europe, as far as we know. Further work on this topic is therefore necessary, particularly since PCR techniques were recommended as diagnostic tools by the OIE in 2018. PCR techniques can currently be used for first intention testing (test performed prior to a confirmatory test), which undoubtedly increases the diversity of decision trees, molecular methods being much more sensitive than traditional techniques (FAT & RTCIT) that used to be commonly summarised based on first FAT then RTCIT, or FAT then PCR, or FAT then RTCIT then PCR.

Some limits of the interpretation of our study have to be pointed out. First of all, regarding the participants themselves, participation was limited to only one laboratory per country to ensure equity in test participation with limited material and capacity. In Europe, the organisation of diagnostic laboratory networks is mainly structured geographically, with several regional laboratories coordinated by national laboratories. Only the national laboratories took part in this exercise, which for the majority was used to either obtain or justify the maintenance of their ISO 17025 accreditation. As far as we know in Europe, National Reference Laboratories carry out most diagnostic analyses for rabies. Some (relatively few) countries have a network of regional laboratories. For those countries with regional laboratories, the NRLs are in charge of organising annual proficiency tests for their national networks. As only national laboratories took part in the ANSES-organised proficiency testing, we could therefore expect a higher level of performance than at regional level. Moreover, the samples were probably not processed under classical test conditions, the “pressure” for success being at its maximum during the test. For this reason, the failure rate of this study was no doubt lower than the failure rate under “standard” diagnostic conditions.

In addition, there is a disadvantage to the fact that the samples to be diagnosed were obtained following experimental infection and therefore known to be either diseased or healthy, as the level of infection is higher than what would be encountered with samples from the field to diagnose. The expected failure rate in this study was therefore most probably lower than what the failure rate under usual laboratory conditions would be. Indeed, samples from the field can be from species less sensitive to the virus, or from animals at an early stage of infection. In the field, the conditions of animal storage prior to brain collection or deterioration of the virus may lead to samples with only a weak positive signal, while we used lyophilised fresh samples to ensure the stable transport conditions required for participating laboratories to receive comparable items. This is all the more so for FAT and RTCIT techniques, when virus antigens are detected by fluorescence. PCR techniques would be less prone to this kind of bias since even on highly degraded samples the RNA remains present for a long time so the samples remain positive [63,64]. On the other hand, for such PCR techniques, highly contaminated samples might increase the risk of cross-contamination inducing false positive results. This was frequently observed during the study, and is revealed by the fact that the lowest specificity was obtained using PCR techniques.

Finally, to reproduce usual diagnostic conditions as closely as possible and for reasons of large-scale feasibility, a single panel was sent each year to the participants in order to test all the techniques on the same samples. The laboratories using different techniques that obtained suspicious or discordant results could have been able to repeat their analysis in order to correct them. Such an undesirable situation could be avoided by including samples that could have a different status depending on the technique (for example positive with conventional RT-PCR but negative with FAT). The possibility of producing samples with such characteristics on a large scale without sacrificing either homogeneity or stability is currently being investigated. On the other hand, this will not solve the problem of the correction of doubtful technical situations such as FAT- or RTCIT-positive but conventional RT-PCR- or real-time RT-PCR-negative cancelling the veracity of the results and therefore potentially leading the laboratory to re-analyse the sample. Indeed, apart from mismatches between particular lyssavirus species and the probe leading to a false negative result for TaqMan RT-PCR, there is little chance that the most sensitive techniques will give negative results when less sensitive techniques are positive.

In this study, we shared the results of performance tests carried out annually using four diagnostic techniques (FAT, RTCIT, conventional RT-PCR and real-time RT-PCR) and with an overall diagnostic conclusion involving the participation of 73 laboratories over almost 10 years. Although the study describes some biases which could underestimate the failure rate, it provides a picture of the success rate, sensitivity and specificity of a large panel of laboratories. From a global epidemiological perspective, such an estimation gives a more realistic idea of the precision of diagnosis than figures provided by the technical evaluation of a single laboratory.

The regular organisation of performance tests has led to an increase in participating laboratory performance over time. Facing a worldwide increase in the number of laboratories that wish to manage their diagnostic activity as part of an accredited quality assurance approach, leading to an increased demand from participants to take part in such proficiency testing, and given that the availability of materials is limited, the demand for the international organisation of such rabies diagnosis proficiency tests is real. The regular organisation of one exercise per continent could meet this need. In such organisation situation, it would also be preferable to select strains used in the panel in regards to the local epidemiological situation. It is of crucial importance to maintain a high-quality rabies diagnosis capability worldwide so as to better detect and therefore react quickly, offering increased disease control capacity which, in the absence of detection and access to treatment, remains fatal for at least 59,000 people every year. Substantial efforts will be needed in the future to develop and maintain such proficiency testing over a broader geographical area.

## Supporting information

S1 Table Virus batches and original strains used in each annual session of the study. (DOCX)

S2 Table Original strain characteristics used in the study. (DOCX)
