# Supplementary material for: Multi-annual performance evaluation of laboratories in post-mortem diagnosis of animal rabies: Which techniques lead to the most reliable results in practice?
Source: PLoS Negl Trop Dis. 2021 Feb 5;15(2):e0009111. doi: 10.1371/journal.pntd.0009111 (PMC7891719; doi:10.1371/journal.pntd.0009111)
Supplement: S1 Table — (DOCX) [file pntd.0009111.s001.docx]

S1 Table: Virus batches and original strains used in each annual session of the study

| Species | Strain origin name | Batch number | 2009 | 2010 | 2011 | 2012 | 2013 | 2014 | 2015 | 2016 | 2017 | 2019 |
| --- | --- | --- | --- | --- | --- | --- | --- | --- | --- | --- | --- | --- |
| ABLV | 96/0648 | ABLV0613 |  |  |  |  |  |  |  |  | x |  |
| ABLV | 96/0648 | ABLV0617 |  |  |  |  |  |  |  |  |  | x |
| ABLV | 96/0648 | ABLV10 |  | x |  |  |  |  |  |  |  |  |
| ABLV | 96/0648 | ABLV11 |  |  | x |  |  |  |  |  |  |  |
| ABLV | 96/0648 | ABLV3012 |  |  |  |  | x |  |  |  |  |  |
| BBLV | 127900 | BBLV0215 |  |  |  |  |  |  | x | x |  |  |
| DUVV | 96132 | DUVV0212 |  |  |  | x |  |  | x |  |  |  |
| DUVV | 96132 | DUVV0511 |  |  |  |  |  |  |  |  |  | x |
| EBLV-1 | 121411 | EBLV109 | x |  |  |  |  |  |  |  |  |  |
| EBLV-1 | 121411 | EBLV1b3112 |  |  |  |  | x |  |  |  |  |  |
| EBLV-1 | 122938 | EBLV110 |  | x |  |  |  |  |  |  |  |  |
| EBLV-1 | 122938 | EBLV111 |  |  | x |  |  |  |  |  |  |  |
| EBLV-1 | 122938 | EBLV1a0814 |  |  |  |  |  | x | x |  |  |  |
| EBLV-1 | 122938 | EBLV1a1214 |  |  |  |  |  |  |  |  | x |  |
| EBLV-1 | 122938 | EBLV1a2012 |  |  |  |  | x |  |  |  |  |  |
| EBLV-1 | 123008 | EBLV1b1014 |  |  |  |  |  |  |  | x |  |  |
| EBLV-1 | 123008 | EBLV1b1918 |  |  |  |  |  |  |  |  |  | x |
| EBLV-1 | EBL1ES10-11 | EBLV11311 |  |  |  | x |  |  |  |  |  |  |
| EBLV-2 | RV1332 | EBLV20612 |  |  |  |  | x |  |  |  |  |  |
| EBLV-2 | RV1332 | EBLV209 | x |  |  |  |  |  |  |  |  |  |
| EBLV-2 | RV1332 | EBLV210 |  | x |  |  |  |  |  |  |  |  |
| EBLV-2 | RV1332 | EBLV211 |  |  | x |  |  |  |  |  |  |  |
| EBLV-2 | RV1332 | EBLV21109 |  |  |  | x |  |  |  |  |  |  |
| EBLV-2 | RV1787 | EBLV20115 |  |  |  |  |  |  | x | x |  |  |
| EBLV-2 | RV1787 | EBLV20516 |  |  |  |  |  |  |  |  | x |  |
| EBLV-2 | RV1787 | EBLV21313 |  |  |  |  |  | x |  |  |  |  |
| NEGATIVE | fox brain origin | NEG07121 |  |  |  | x |  |  |  |  |  |  |
| NEGATIVE | fox brain origin | NEG07122 |  |  |  | x |  |  |  |  |  |  |
| NEGATIVE | fox brain origin | NEG07123 |  |  |  | x |  |  |  |  |  |  |
| NEGATIVE | fox brain origin | NEG091 | x |  |  |  |  |  |  |  |  |  |
| NEGATIVE | fox brain origin | NEG092 | x |  |  |  |  |  |  |  |  |  |
| NEGATIVE | fox brain origin | NEG1001 |  | x |  |  |  |  |  |  |  |  |
| NEGATIVE | fox brain origin | NEG1002 |  | x |  |  |  |  |  |  |  |  |
| NEGATIVE | fox brain origin | NEG1003 |  | x |  |  |  |  |  |  |  |  |
| NEGATIVE | fox brain origin | NEG1101 |  |  | x |  |  |  |  |  |  |  |
| NEGATIVE | fox brain origin | NEG1102 |  |  | x |  |  |  |  |  |  |  |
| NEGATIVE | fox brain origin | NEG3812 |  |  |  |  | x |  |  | x |  |  |
| NEGATIVE | fox brain origin | NEGATIF1713 |  |  |  |  |  | x |  |  |  |  |
| NEGATIVE | fox brain origin | NEGATIF17131 |  |  |  |  |  |  | x |  |  |  |
| NEGATIVE | fox brain origin | NEGATIF17132 |  |  |  |  |  |  | x |  |  |  |
| NEGATIVE | fox brain origin | NEGFOX1118 |  |  |  |  |  |  |  |  |  | x |
| NEGATIVE | pig brain origin | NEGPIG1618 |  |  |  |  |  |  |  |  |  | x |
| RABV | 124155 | DOG1217 |  |  |  |  |  |  |  |  |  | x |
| RABV | 124155 | RABVmo1715 |  |  |  |  |  |  |  | x |  |  |
| RABV | Ariana 1991 | ARIANA09 | x |  |  | x |  |  |  |  |  |  |
| RABV | Ariana 1991 | ARIANA09W | x |  |  |  |  |  |  |  |  |  |
| RABV | CNVivEst1012 | CnViV1513 |  |  |  |  |  | x |  |  |  |  |
| RABV | CNVIVEst1012 | CnVivEST2712 |  |  |  |  | x |  |  |  |  |  |
| RABV | CnvivPologne | RACCOON09 | x |  |  |  |  |  |  |  |  |  |
| RABV | GR36_12 | GR0315 |  |  |  |  |  |  |  | x |  |  |
| RABV | GR36_12 | GREECE0315 |  |  |  |  |  |  | x |  |  |  |
| RABV | GS7_1_11 | GS1300214 |  |  |  |  |  |  |  | x |  |  |
| RABV | GS7_1_11 | GS51517 |  |  |  |  |  |  |  |  |  | x |
| RABV | GS7_1_11 | GS709 | x |  |  |  |  |  |  |  |  |  |
| RABV | GS7_1_11 | GS710 |  | x |  |  |  |  |  |  |  |  |
| RABV | GS7_1_11 | GS711 |  |  | x |  |  |  |  |  |  |  |
| RABV | GS7_1_11 | GS711100 |  |  | x |  |  |  |  |  |  |  |
| RABV | GS7_1_11 | GS71115 |  |  |  |  |  |  |  | x | x |  |
| RABV | GS7_1_11 | GS7118 |  |  | x |  |  |  |  |  |  |  |
| RABV | GS7_1_11 | GS71201510 |  |  |  | x |  |  |  |  |  |  |
| RABV | GS7_1_11 | GS713014 |  |  |  |  |  | x |  |  |  |  |
| RABV | GS7_1_11 | GS715014 |  |  |  |  |  | x |  |  |  |  |
| RABV | GS7_1_11 | GS71510 |  |  |  | x |  |  |  |  |  |  |
| RABV | GS7_1_11 | GS71813 |  |  |  |  |  | x | x |  |  |  |
| RABV | CVS27 | CVS270113 |  |  |  |  |  | x |  |  |  |  |
| RABV | CVS27 | CVS270417 |  |  |  |  |  |  |  |  |  | x |
| RABV | CVS27 | CVS270616 |  |  |  |  |  |  |  |  | x |  |
| RABV | CVS27 | CVS2711 |  |  | x |  |  |  |  |  |  |  |
| RABV | CVS27 | CVS271312 |  |  |  |  | x |  |  |  |  |  |
| RABV | CVS27 | CVS271314 |  |  |  |  |  |  | x | x |  |  |
| RABV | RABVdogES11-11 | Dog1911 |  |  |  | x |  |  |  |  |  |  |
| RABV | RABVdogES11-11 | DogEs1911 |  |  |  |  | x |  |  |  |  |  |
| RABV | RABVdogES11-11 | RABVdog1215 |  |  |  |  |  |  |  |  | x |  |
| RABV | RABVMac37-12 | RABVMAC1013 |  |  |  |  |  | x |  |  |  |  |
| RABV | Slovaquie13-16 | RABVfox0217 |  |  |  |  |  |  |  |  |  | x |
