# Supplementary material for: Multi-annual performance evaluation of laboratories in post-mortem diagnosis of animal rabies: Which techniques lead to the most reliable results in practice?
Source: PLoS Negl Trop Dis. 2021 Feb 5;15(2):e0009111. doi: 10.1371/journal.pntd.0009111 (PMC7891719; doi:10.1371/journal.pntd.0009111)
Supplement: S2 Table — (DOCX) [file pntd.0009111.s002.docx]

S2 Table : Original strain characteristics used in the study

| Species | Strain name | Species originaly  infected | Date of  isolation | Site of  isolation | Country |
| --- | --- | --- | --- | --- | --- |
| ABLV | 96/0648 | *Pteropus alecto* | 1997 | ND | Australia |
| BBLV | 127900 | *Myotis nattrreri* | 23/07/2012 | Hemilly | France |
| DUVV | 96132 | *Human Brain* | 1971 | ND | South Africa |
| EBLV-1 | 121411 | *Eptesicus serotinus* | 25/09/2000 | Fouesnant | France |
| EBLV-1 | 122938 | *Eptesicus serotinus* | 26/08/2002 | Gueret | France |
| EBLV-1 | 123008 | *Eptesicus serotinus* | 06/09/2002 | Lurcy Levis | France |
| EBLV-1 | EBL1ES10-11 | *Eptesicus isabellinus* | Jun-85 | Huelva | Spain |
| EBLV-2 | RV1332 | *Myotis daubentonii* | 01/09/2002 | Lancashire | United Kindom |
| EBLV-2 | RV1787 | *Myotis daubentonii* | 01/09/2004 | Surrey | United Kindom |
| RABV | 124155 | *Canis lupus familiaris* | 24/05/2004 | Saint-Gery | France via Morocco |
| RABV | Ariana 1991 | *Canis lupus familiaris* | 1991 | ND | Tunisia |
| RABV | CNVivEst1012 | *Nyctereutes procyomoïdes* | 05/05/2006 | ND | Estonia |
| RABV | CnvivPologne | *Nyctereutes procyomoïdes* | 1985 | ND | Poland |
| RABV | GR36_12 | *Vulpes vulpes* | 10/2012 | Palaiokastro | Greece |
| RABV | GS7_1_11 | *Vulpes vulpes* | 1986 | Nord-Est | France |
| RABV | CVS27 | laboratory fixed strain |  |  |  |
| RABV | RABVdogES11-11 | *Canis lupus familiaris* | 07/04/2010 | Melilla | Spain |
| RABV | RABVMac37-12 | *Vulpes vulpes* | nov.-11 | ND | North Macedonia |
| RABV | Slovaquie13-16 | *Vulpes vulpes* | 2015 | Stara Lubovna | Slovakia |
